# Physiological Effects of Visual Stimulation by a Japanese Low Wooden Table: A Crossover Field Experiment

**DOI:** 10.3390/ijerph20146351

**Published:** 2023-07-12

**Authors:** Harumi Ikei, Hyunju Jo, Yoshifumi Miyazaki

**Affiliations:** Center for Environment, Health and Field Sciences, Chiba University, 6-2-1 Kashiwa-no-ha, Chiba 277-0882, Japan; hyunju.jo@chiba-u.jp (H.J.); ymiyazaki@faculty.chiba-u.jp (Y.M.)

**Keywords:** nature therapy, wooden table, visual stimulation, prefrontal cortex activity, autonomic nervous activity, near-infrared spectroscopy, heart rate variability, physiological relaxation, psychological benefit, field study

## Abstract

The purpose of this study was to evaluate the physiological effects of visual stimulation by a unique Japanese low wooden table on the prefrontal cortex and autonomic nervous activities. A within-participants experiment with 26 male university students was conducted in a Japanese-style room. The visual stimuli were a low wooden table (WT) made of Japanese cypress and a low cloth-covered table (control) for an exposure time of 90 s. Near-infrared spectroscopy was used to measure the prefrontal cortex activity in the left and right prefrontal cortices as an indicator of oxyhemoglobin (oxy-Hb) concentration. Autonomic nervous activity was measured as an indicator of sympathetic (low-frequency/high-frequency component ratio, LF/HF), and parasympathetic (high-frequency components, HF) nervous activities were assessed by heart rate variability. Furthermore, the modified semantic differential method and the Profile of Mood States 2nd edition were used to measure psychological responses. Physiologically, the oxy-Hb concentration in the left prefrontal cortex and ln (LF/HF) were significantly lower during visual exposure to the WT than to the control. Psychologically, more comfortable, relaxed, and natural impressions, as well as improved mood states, were reported during visual stimulation to the WT than to the control. This study demonstrated that viewing a WT led to physiological relaxation and had a positive psychological effect on the participants.

## 1. Introduction

Research on human comfort in the indoor environment has focused on eliminating factors that can negatively affect humans, such as heat, air quality, sound, and light [1,2,3]. In recent years, there has been growing interest in research around promoting positive effects, such as improved quality of life, in the indoor environment. Studies focusing on well-being are beginning to be conducted [4]. Wood is one of the natural materials often used in indoor environments. In various buildings, such as houses, offices, and public facilities, it is used not only as a structural material, but also as an interior finishing material that is directly seen and touched. It is known that contact with wood imparts positive impressions, such as comfort and attractiveness, to people [5,6]. On the contrary, not much data on the physiological effects using physiological indices have been accumulated [7,8,9]; however, with the recent establishment of physiological measurement technology, data on the brain, autonomic nervous, endocrine, and immune activity of humans induced by wood-derived stimuli have been accumulated, mainly through studies conducted in Japan [10].

Previous studies on adult participants have conducted, using simultaneous measurements of brain and autonomic nervous activity in an artificial climate chamber with adjustable temperature, humidity, and soundproof functions [11,12,13,14,15,16,17,18,19,20,21,22]. Regarding the physiological effects of wood-derived visual stimuli on humans, wooden wall image experiments using large-scale displays capable of presenting realistic visual stimuli have been conducted [11,12]. Nakamura et al. [11] used two types of wall images with laminar arranged vertically or horizontally to investigate the effects on brain and autonomic nervous activity in young women in their 20 s. The results showed that wooden wall image-based visual stimulation for 90 s significantly decreased the concentration of oxyhemoglobin (oxy-Hb) in the left and right prefrontal cortices relative to that when viewing a gray image (control). Ikei et al. [12] used two types of wall images composed of knotty or clear wood, respectively, to also study the effects on brain and autonomic nervous activity in female participants in their 20 s. The results showed that (1) visual stimulation by the knotty wood image was associated with a significant decrease in oxy-Hb concentration in the right prefrontal cortex and significant enhancement of parasympathetic nervous activity, and (2) visual stimulation by a clear wood image was associated with a significant decrease in oxy-Hb concentration in the left prefrontal cortex and significant suppression of sympathetic nervous activity. Studies on visual stimulation with wooden wall images have confirmed that stimulation is associated with a physiologically relaxing effect, as shown by calmed prefrontal cortex activity, increased parasympathetic nervous activity, and suppressed sympathetic nervous activity. Experiments with olfactory [13,14,15,16] and tactile stimuli [17,18,19,20,21,22] have also been conducted. The results also showed that similar to visual stimuli results, a series of physiologically relaxing effects, such as calmed prefrontal cortex activity and enhanced parasympathetic nervous activity, were heightened during relaxation, and that suppressed sympathetic nervous activity was heightened during stress. Thus, in laboratory experiments, several studies have examined the effects of wood-derived visual, olfactory, and tactile stimuli on human physiological responses; however, studies targeting actual buildings are lacking.

In recent years, the utilization of wood has also attracted attention from the perspective of the Sustainable Development Goals (SDGs) [23]. “Goal 15: Using wood produced through sustainable forest management” is directly connected to “Goal 12: Responsibility to Produce and Use”, which calls for ensuring sustainable consumption and consumption patterns. Wood requires less energy to manufacture and process than other materials [24] and stores carbon when used as a construction and furniture material [25]. Therefore, wood also functions as long-term storage of carbon from atmospheric CO_2_, a greenhouse gas, originally absorbed by trees while they grew [26].

In Japan, mainly from the standpoint of fire protection, the use of wood has been restricted depending on the building’s size, use, and location. However, in recent years, technological developments regarding wood fire-resistant materials and cross-laminated timber have led to the promotion of wood in the construction of mid- and high-rise buildings [27]. The office building “Mokuzai Kaikan” consists of seven stories aboveground and one story underground and was built in Shinkiba (Koto, Tokyo) in 2009. More than 1000 m^3^ of Japanese lumber, mainly Japanese cypress wood, a typical Japanese conifer, was used for the building’s interior, exterior, and structural components [28]. The building, which combines traditional Japanese wooden construction methods with the latest technology, including wood fire-resistant materials, has attracted widespread attention and received the Annual Architectural Design Commendation 2011 and the Japan Institute of Architects Award in 2010, among others. In addition to office space, Mokuzai Kaikan also has halls, conference rooms, and Japanese-style rooms as elements of traditional Japanese wooden architecture. The prototype of a modern Japanese-style room was established approximately 700 years ago and has since been used as a familiar living space for Japanese people [29]. A Japanese-style room’s interior is composed of natural materials, such as wood, and the floor is covered entirely with grass mats called tatami, which are generally stepped on only by bare feet. The table is a low wooden table called zataku [30]. Since World War II, along with rapid economic growth, Japanese lifestyles have become Westernized and have changed from sitting on the floor to sitting on chairs. However, according to a floor plan survey conducted on single-family families in their 20–40 s, the adoption rate of tatami in new construction was 74.7% in 2016, equating to nearly three-quarters of the total number of Japanese-style rooms introduced that year [31]. This finding is partly explained by the idea that the Japanese-style room is deeply rooted in Japanese culture. However, no studies on field experiments in wooden spaces have been conducted to clarify the effects of wood-derived visual stimuli, such as a Japanese low wooden table, on human brain and autonomic nervous activity.

In previous laboratory experiments, various wood-derived stimuli were reported to calm prefrontal cortex activity, increase parasympathetic nervous activity (reflecting a state of relaxation), and suppress sympathetic nervous activity (reflecting a state of stress) [11,12,13,14,15,16,17,18,19,20,21,22]. Therefore, in the present study, a field experiment was performed using these physiological indices to clarify the physiological effects of the visual stimulation by a low wooden table installed in a Japanese-style room in Mokuzai Kaikan.

## 2. Materials and Methods

### 2.1. Participants

**Figure 1:**
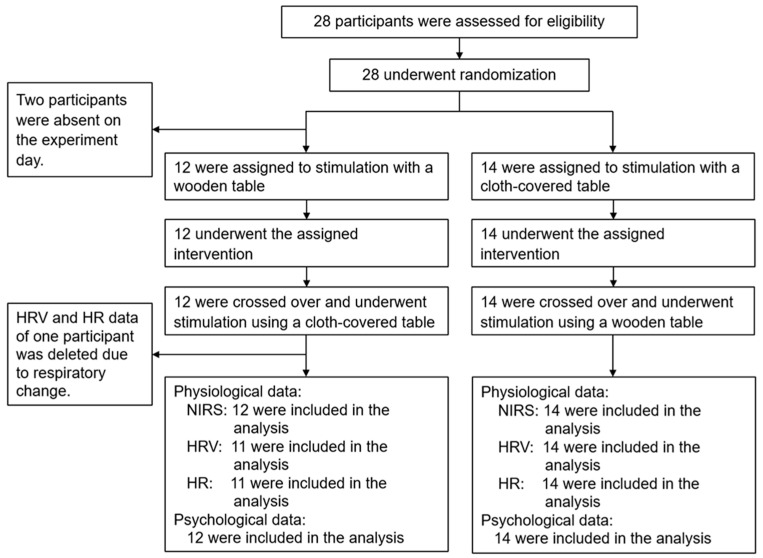
Flowchart of the experiment showing the participant screening, enrollment, follow-up, and analysis flow. NIRS, near-infrared spectroscopy; HRV, heart rate variability; HR, heart rate.

Since sex-based differences are expected with regard to physiological responses, only the male sex was selected this time. Japanese male undergraduates and graduates participated in the study. Regarding the sample size, based on the effect size obtained from the laboratory data collected by our team when previously investigating the effects of wood-derived visual stimuli [11,12], G*Power software (version 3.1; Heinrich Heine Universität Düsseldorf, Germany) with an α error of 0.05 and a power of 0.8 calculated that the required sample size was between 10 and 25 participants. Initially, 28 students participated, but two were unable to attend on the study day, which resulted in a final total of 26 male students. This study used a randomized block design. The participants were assigned to one of the two intervention groups that received different orders of visual stimulation (Figure 1). The inclusion criteria were as follows: Potential subjects without chronic rhinitis, asthma, or arrhythmias and non-habitual smokers, and a visual acuity based on Landolt ring vision (1.0 equals 20/20) [32] >0.3 (including correction by contact lens or glasses, if worn). Table 1 presents the physical characteristics of the participants. The body temperature of each participant was measured on the day of the experiment. Before conducting the study, all participants were sufficiently informed about the study aim and procedures, following which the participants provided written consent to participate. The study was conducted in accordance with the guidelines of the Declaration of Helsinki, and the protocol of the experiment was approved by the Ethics Committee of the Center for Environment, Health and Field Sciences, Chiba University, Japan (approval number: 56) and was registered in the University Hospital Medical Information Network of Japan (ID: UMIN000047569).

### 2.2. Visual Stimulation

**Figure 2:**
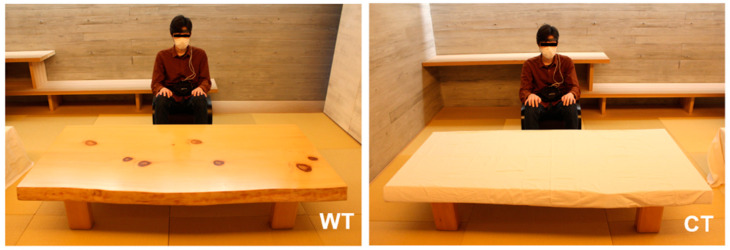
Scenery of visual exposure. WT, a low wooden table made of Japanese cypress (*Chamaecyparis obtusa*); CT, a low cloth-covered table of the same size as the WT.

Figure 2 shows the scenery used for visual exposure. Two similarly shaped tables were prepared, one for wood-derived visual stimulation and the other for control. The visual stimulus was a low wooden table (WT) made of Japanese cypress (*Chamaecyparis obtusa*) from Kiso, Nagano Prefecture, Japan. Japanese cypress, one of the representative precious woods in Japan [29], is widely used as the tabletop of low tables. The WT size was 1815 mm long × 875 mm wide × 380 mm high (Figure 1 left). The control was a low table of the same size covered with a white cotton cloth (CT) (Figure 1, right). The distance from the participant to the visual stimuli was approximately 100 cm.

### 2.3. Physiological Measurement

#### 2.3.1. Near-Infrared Spectroscopy (NIRS)

The changes in oxy-Hb concentrations in the left and right prefrontal cortices detected by NIRS were used as a parameter of brain activity. The regional cerebral blood flow associated with focal neural activity in the brain correlates with hemoglobin levels (oxyhemoglobin and total hemoglobin) using NIRS [33]. This tool finds extensive application as a modality for gauging brain activity across a spectrum of research domains spanning such fields as medicine, psychology, and social sciences [34,35].

A portable NIRS device with two channels (Pocket NIRS Duo, DynaSense, Hamamatsu, Japan [36]) was employed. Two NIRS probes were affixed symmetrically on the participant’s bilateral forehead. Oxy-Hb concentrations in the prefrontal cortex were recorded at an interval of 1 s during rest and exposure periods. The data for the average 90 s of exposure to the visual stimulus were calculated as the difference from the average 30 s rest period before visual exposure.

#### 2.3.2. Heart Rate Variability (HRV) and Heart Rate (HR)

HRV and HR were used as the autonomic nervous system parameters [37,38]. HRV was analyzed for the periods between consecutive R waves. A portable electrocardiogram (Activtracer AC-301A; GMS, Tokyo, Japan) was used to record electrocardiography. The power of the high-frequency (HF; 0.15–0.40 Hz) and low-frequency (LF; 0.04–0.15 Hz) components of HRV were calculated using the maximum entropy method (MemCalc/Win; GMS, Tokyo, Japan) [39,40]. The HF component indicates the activity of the parasympathetic nervous system, while the LF/HF ratio indicates the activity of the sympathetic nervous system. In this study, the natural logarithmic values of HF (ln (HF)) and the LF/HF ratio (ln (LF/HF)) were used to normalize the participants’ individual HRV values [41]. Similar to NIRS, the data for the average 90 s of exposure to the visual stimulus were calculated as the difference from the average 30 s rest period before visual exposure.

Furthermore, respiratory changes can influence HRV data. Thus, the participants’ respiratory rates were estimated during the period between the two stimuli. The respiratory rate can be estimated from the HRV power spectrum [42]. Generally, HR accelerates during inspiration and decelerates during expiration [43,44]; thus, the respiratory rate can be estimated from the HF component’s dominant frequency. The maximum entropy method was used to calculate the HRV power spectrum and the associated frequency as the dominant respiratory frequency during the measurement period was used to locate the HF component’s maximum power. To detect the HF component’s peak frequency, the model order for spectral analysis was chosen from the seventh to twelfth orders, with the ninth order used in principle.

### 2.4. Psychological Measurement

The impression and mood states evoked by the visual stimuli were assessed via questionnaires using a modified semantic differential (SD) method [45] and the short version of the Profile of Mood States 2nd edition (POMS 2) [46,47]. The modified SD method uses three opposing adjective pairs (comfortable–uncomfortable, relaxed–awakening, natural–artificial) to subjectively assess the impression on a 13-point scale. The POMS 2 short version accessed mood state via 35 questions with five negative mood state subscales (anger–hostility (A–H), confusion–bewilderment (C–B), depression–dejection (D–D), fatigue–inertia (F–I), and tension–anxiety (T–A)), as well as two positive mood state subscales (vigor–activity (V–A) and friendliness (F)). Furthermore, the total mood disturbance (TMD) score was analyzed using a determined formula ((A–H) + (C–B) + (D–D) + (F–I) + (T–A) − (V–A)). A small score for TMD indicates a positive mood state.

The data collected using the questionnaire were scored based on the responses of the participants (13 levels for the modified SD method (−6 to 6) and five levels for the POMS 2 (0 to 4)), and the average value was calculated.

### 2.5. Study Protocol

**Figure 3:**
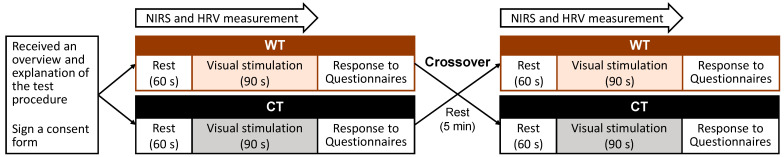
The measurement protocol for visual exposure to the low wooden table (WT) vs. the low cloth-covered table (CT). The exposure order of the WT and CT was counterbalanced. The study employed a within-participants design.

The experiment was performed in a Japanese-style room at Mokuzai Kaikan of Tokyo Mokuzai Tonya Kyoudou Kumiai (Shinkiba, Koto, Tokyo). The room’s temperature, relative humidity, and illumination were 22.8 ± 0.4 °C, 54.0% ± 8.3%, and 130–140 lx, respectively. The measurement protocol is shown in Figure 3. The participants received an overview and explanation of the test procedure in the waiting room, signed a consent form, and were then transferred to the experimental room. Before the participant entered the room, the WT and CT were covered with a white cloth. The white cloth that covered both tables was made of the same material as the one used for the CT. After entering the room, the participant sat on a chair in front of the table. The experimenter fitted the physiological sensors and gave a detailed description of the experimental procedure. First, the participant rested for 60 s while looking at the table covered with the white cloth. The experimenter then removed the cloth, and the participant was exposed to either the WT or CT for 90 s. During the period of rest and exposure, NIRS, HRV, and HR data were continuously recorded. After completion of the exposure, the SD and POMS 2 self-reported questionnaires were filled in. During the five-minute resting period between the two experimental blocks, the participant sat in a low chair in a relaxed resting state. The order of exposure was counterbalanced.

### 2.6. Statistical Analysis

SPSS (version 21.0, IBM SPSS Statistics for Windows; IBM Corp., Armonk, NY, USA) was used to perform statistical analysis. *p*-values of <0.05 were considered to be indicative of statistical significance.

Paired *t*-tests were used to compare the differences in the NIRS, HRV, and HR physiological data and respiratory rate between the WT (exposure period (90 s) minus the rest period (30 s)) and CT (exposure period (90 s) minus rest period (30 s)). Cohen’s *d* (*d*) [48] was calculated as the effect size for the physiological indices. The Wilcoxon signed-rank test was performed to compare the differences in the psychological data of the modified SD method and POMS 2 between the WT and CT. The probability of superiority (*PS_dep_*) [49] was calculated as the effect size of each psychological index.

## 3. Results

### 3.1. Physiological Effects

#### 3.1.1. NIRS

**Figure 4:**
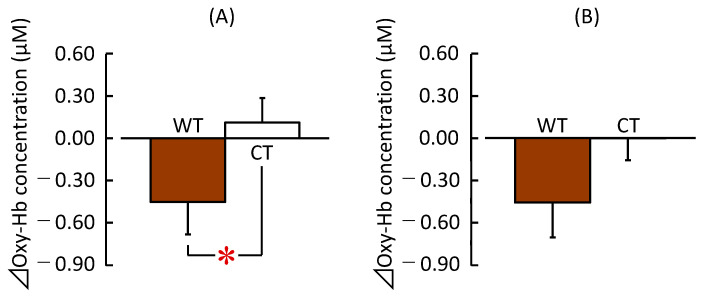
⊿Oxy-Hb concentrations in the prefrontal cortex during visual exposure to the low wooden table (WT) vs. low cloth-covered table (CT) for 90 s. (**A**) Changes in the oxy-Hb concentration in the left prefrontal cortex. (**B**) Changes in the oxy-Hb concentration in the right prefrontal cortex (*n* = 26, mean ± standard error). * *p* < 0.05 (WT vs. CT), paired *t*-test.

Figure 4 depicts the results of the left and right prefrontal activities. Figure 4A shows the average ⊿oxy-Hb concentrations in the left prefrontal cortex during 90 s visual exposures to WT and CT. Visual exposure to WT significantly decreased the ⊿oxy-Hb concentrations relative to those during visual exposure to the CT (WT: −0.45 ± 0.23 μM; (mean ± SE) CT: 0.11 ± 0.17 μM; *t* (25) = −2.490, *p* = 0.020, *d* = 0.49). No significant difference was observed between WT and CT on the ⊿oxy-Hb concentrations in the right prefrontal cortex (WT: −0.46 ± 0.25 μM; CT: −0.00 ± 0.15 μM; *t* (25) = −1.915, *p* = 0.067, *d* = 0.42). Thus, the findings indicated that the left prefrontal cortex activity was lower during visual exposure to the WT than during visual exposure to the CT.

#### 3.1.2. HRV and HR

One participant who showed a significant change in respiratory rate while exposed to visual stimuli was excluded because variations in these values could influence the HRV data. No significant difference was noted in the respiratory rate between the WT and CT data; therefore, statistical analysis of the HRV and HR data was performed.

**Figure 5:**
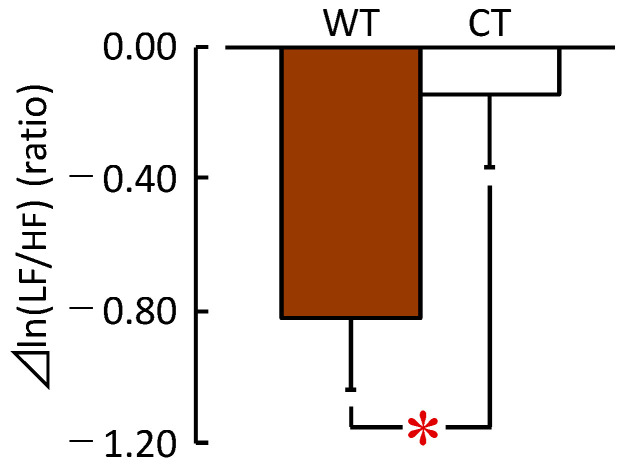
Changes in the ⊿ln (LF/HF) ratio, which reflects the sympathetic nervous activity during visual exposure to the low wooden table (WT) vs. the low cloth-covered table (CT) for 90 s. (*n* = 25, mean ± standard error). * *p* < 0.05, paired *t*-test.

The HRV data revealed a significant difference in the sympathetic nervous activity in response to exposure to WT and CT. Figure 5 shows the average ⊿ln (LF/HF) ratio during the 90 s visual exposure to WT and CT. The ⊿ln (LF/HF) ratios were −0.82 ± 0.22 during the exposure to the WT and −0.14 ± 0.22 during exposure to the CT. This result indicates that the sympathetic nervous activity was significantly lower during exposure to the WT than the CT (*t* (24) = −2.152, *p* = 0.042, *d* = 0.62).

There was no observed significant difference between WT and CT in the ⊿ln (HF), which reflects the parasympathetic nervous activity (WT: 0.17 ± 0.11 ms^2^; CT: −0.01 ± 0.10 ms^2^; *t* (24) = 1.265, *p* = 0.218, *d* = 0.35). Furthermore, there was no significant difference in ⊿HR while viewing the WT and CT (WT: −0.12 ± 0.72 beats/min; CT: 0.29 ± 0.51 beats/min; *t* (24) = −0.608, *p* = 0.549, *d* = 0.13).

### 3.2. Psychological Effects

#### 3.2.1. Modified SD Method

**Figure 6:**
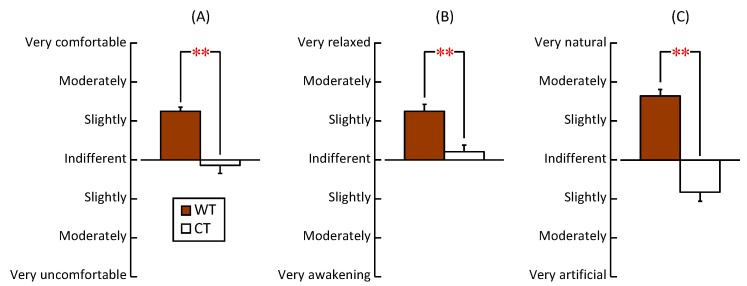
The modified SD method was used to evaluate the psychological effects based on three opposing adjective pairs after viewing the low wooden table (WT) vs. the low cloth-covered table (CT). (**A**) Comfortable vs. uncomfortable. (**B**) Relaxed vs. awakening. (**C**) Natural vs. artificial (*n* = 26, mean ± standard error). ** *p* < 0.01 (WT vs. CT). Wilcoxon signed-rank test.

Figure 6 summarizes the results of evaluating the subjective feelings regarding the three subscales measured by the modified SD method after viewing the WT and CT. On the comfortable to uncomfortable subscale, the participants reported having slightly-to-moderately comfortable impressions of the WT but reported almost indifferent impressions of the CT (Figure 6A; *p* < 0.001, *PS_dep_* = 0.846). On the subscale of relaxed to awakening, the participants reported that WT evoked a slightly-to-moderately relaxed impression, but CT evoked an indifferent-to-slightly relaxed impression (Figure 6B; *p* < 0.001, *PS_dep_* = 0.769). On the natural to artificial subscale, the participants perceived that the WT induced a slightly-to-moderately natural impression, whereas CT evoked an indifferent-to-slightly artificial impression (Figure 6C; *p* < 0.001, *PS_dep_* = 1.000). These results indicate that more comfortable, relaxed, and natural impressions were evoked by viewing the WT than by viewing the CT.

#### 3.2.2. Profile of Mood State 2nd Edition (POMS 2)

**Figure 7:**
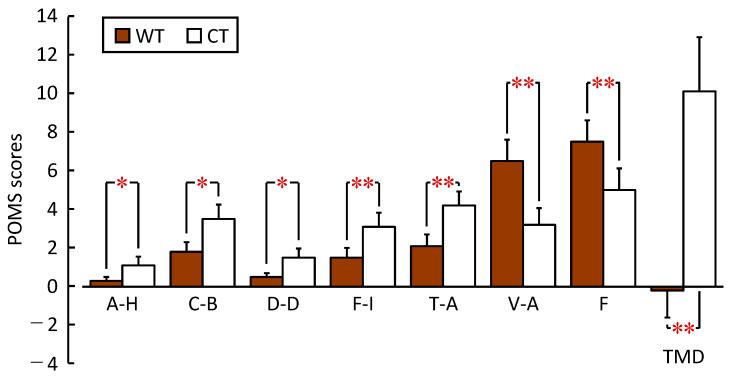
Psychological effects were evaluated by POMS 2 after viewing the low wooden table (WT) and low cloth-covered table (CT) (*n* = 26, mean ± standard error). ** *p* < 0.01, * *p* < 0.05 (WT vs. CT), Wilcoxon signed-rank test. A–H, anger–hostility; C–B, confusion–bewilderment; D–D, depression–dejection; F–I, fatigue–inertia; T–A, tension–anxiety; V–A, vigor–activity; F, friendliness; TMD, total mood disturbance.

Figure 7 summarizes the POMS 2 evaluation results of the subjective mood states after viewing the WT and CT. Regarding the negative mood state subscale, the mean scores of A–H, C–B, and D–D were significantly lower after viewing the WT than after viewing the CT at *p* < 0.05 (A–H (WT: 0.4 ± 0.2; CT: 1.1 ± 0.5, *p* = 0.047, *PS_dep_* = 0.231), C–B (WT: 1.9 ± 0.5; CT: 3.5 ± 0.8, *p* = 0.043, *PS_dep_* = 0.500), D–D (WT: 0.5 ± 0.2; CT: 1.5 ± 0.5, *p* = 0.023, *PS_dep_* = 0.346)). Furthermore, the mean scores of F–I and T–A were lower after viewing the WT than for the CT, with a significance rate of *p* < 0.01 (F–I (WT: 1.5 ± 0.5; CT: 3.1 ± 0.7, *p* = 0.001, *PS_dep_* = 0.615); T–A (WT: 2.1 ± 0.6; CT: 4.2 ± 0.7, *p* = 0.007, *PS_dep_* = 0.615)). The positive mood state subscales, such as V–A and F, were significantly higher after viewing the WT than after viewing the CT (V–A (WT: 6.5 ± 1.1; CT: 3.2 ± 0.9, *p* < 0.001, *PS_dep_* = 0.577); F (WT: 7.5 ± 1.1; CT: 5.0 ± 1.1, *p* = 0.001, *PS_dep_* = 0.577)). The TMD score was significantly lower after viewing the WT than after viewing the CT (WT: −0.2 ± 1.4, CT: 10.1 ± 2.8, *p* < 0.001, *PS_dep_* = 0.769).

## 4. Discussion

The purpose of this study was to evaluate the effects of visual stimuli on prefrontal cortex and autonomic nervous activity using a low wooden table in a Japanese-style room in Mokuzai Kaikan. In this study, the physiological effects of visual stimulation in the Japanese-style room at Mokuzai Kaikan on prefrontal cortex and autonomic nervous activity were investigated by measuring oxy-Hb concentration using NIRS and the sympathetic and parasympathetic nervous activity using HRV in young males in their 20 s. Additionally, subjective evaluations of the psychological effects on the study’s participants were obtained to corroborate the physiological responses. The visual stimulation of a low wooden table (WT) in a Japanese-style room for 90 s significantly decreased the oxy-Hb concentration in the left prefrontal cortex and ln (LF/HF) compared to a low table covered with a white cotton cloth (CT; control). The subjective evaluation also significantly increased comfort, relaxation, and nature and improved overall mood compared to the control.

The left prefrontal oxy-Hb concentration and ln (LF/HF), a measure of sympathetic nervous activity, was significantly lower while viewing a WT for 90 s in a Japanese-style room than while viewing the CT. In a previous study on the effects of wood-derived visual stimuli on human physiological responses, Ikei et al. [12] conducted a wooden wall image experiment using a large display in a sound-proofed laboratory with constant temperature, humidity, and illumination for young women in their 20 s. Two types of wooden interior wall images of knotty and clear wood were used, and a gray image was used as the control. The results of the 90 s presentation of the full-scale wooden wall images showed (1) a significant decrease in oxy-Hb concentration (vs. control) in the right prefrontal cortex and a significant increase in parasympathetic nervous activity (vs. pre-measurement values) in the knotty wood image, and (2) a significant decrease in the left prefrontal oxy-Hb concentration (vs. control) and a significant decrease in sympathetic nervous activity (vs. pre-measurement values) in the clear wood image. The left and right prefrontal cortices and sympathetic and parasympathetic nervous activity responses differed depending on the presence or absence of a knot. However, both resulted in a state of relaxation. Nakamura et al. [11] also used the same experimental protocol to study visual stimulation with full-scale wall images composed of knot-free Japanese cedar wood arranged vertically and horizontally; they found that both the vertical and horizontal wall images significantly reduced left and right prefrontal oxy-Hb concentration levels in young women (vs. control). The present field experiment in young men observed a series of physiological relaxing effects shown by calming of left prefrontal cortex activity and suppression of sympathetic nervous activity relative to those while viewing the control. This result is consistent with that of a previous laboratory experiment in young women.

The subjective evaluation results were consistent with brain and autonomic nervous activity. In the impression evaluation using the modified SD method, viewing the WT was rated as significantly more “comfortable”, “relaxing”, and “natural” than viewing the CT that served as the control. In the mood evaluation by POMS 2, the WT had significantly lower negative mood scale scores for “anger–hostility (A–H)”, “confusion–bewilderment (C–B)”, “depression–dejection (D–D)”, “fatigue–inertia (F–I)”, “tension–anxiety (T–A)”, and “total mood disturbance (TMD), as well as significantly higher positive mood scales for “vigor–activity (V–A)” and “friendliness (F)” than the control. The results of this study agree with those of previous studies [11,12,50] on wood mural images performed in laboratory experiments using large displays. In previous studies on actual lumber, such as panels and wood decks, it has been reported that regardless of the presence or absence of knots, lumber with good “harmony” and “activity” properties perceived when looking at the face of the lumber was preferred [51]. Regarding knotty lumber, lumber with high “homogeneity” [52,53] and “evenness” [54] of the knots was preferred. It can be inferred that the WT used in this study received a favorable subjective evaluation because the knots were homogeneous in size and evenly arranged.

Wood is a representative natural material that is friendly and familiar. It is a practical material for stress reduction and SDGs in modern, urbanized, man-made environments. Miyazaki advocated the “back to nature theory” [55,56] regarding the relaxation effect of wood on physiological responses. The theory is that in the stressful situations currently experienced in urban environments, exposure to nature-derived stimuli such as wood relaxes us and brings us physiologically closer to our original state as human beings [56,57,58,59]. Wood has long been used for housing and other construction purposes. In recent years, from the perspective of the SDGs, it is expected that wood will be utilized in mid- and high-rise buildings worldwide, which was not used much in the past [59,60,61]. There is a possibility that wood contributes to stress reduction in people working and living in these buildings; however, more evidence of this possibility is needed.

To the best of our knowledge, no study has examined the effects of visual stimulation by a wooden table in an actual building on brain and autonomic nervous activity thus far. The novelty of this study is that (1) physiological data from participants in experiments performed in Mokuzai Kaikan, a typical Japanese mid-rise building with extensive use of wood, were collected, contributing to this field of research considering that there is a paucity of physiological data from field experiments; (2) the physiological effects of visual stimulation by a wooden table in a Japanese-style room, a typical Japanese living room style, were investigated; (3) the physiological relaxation effect was clarified for the first time through simultaneous measurement of prefrontal cortex activity and sympathetic and parasympathetic nervous activity.

Although scientific data on the effects of wood-derived visual stimuli on human physiological responses have been accumulated through laboratory experiments conducted mainly by Japanese research teams [10], there have been no reports on living spaces with actual wooden elements, so the field is still in its infancy. In the future, it will be necessary to accumulate scientific data by conducting field experiments in offices and residences that are actually in use. This study was conducted with young males in their 20 s. However, to generalize the study findings, results from participants of different genders and ages must be obtained. Furthermore, considering today’s high-stress society, it is necessary to accumulate data on high-stress individuals with various mental illnesses and developmental disabilities.

## 5. Conclusions

Oxy-Hb concentrations in the left prefrontal cortex and sympathetic nervous activity were significantly lower during visual stimulation by a WT (low wooden table) in a Japanese-style room in Mokuzai Kaikan for 90 s than while viewing a CT (low cloth-covered table; control). In addition, the subjective impressions of the participants assessed by the SD method showed significantly more comfortable, relaxed, and natural feelings after viewing the WT than after viewing the CT, which is consistent with the mood ratings by the POMS 2 showing decreased negative mood states and increased positive mood states after viewing the WT. These results agree with the physiological responses observed in the prefrontal cortex and autonomic nervous activity.

## Figures and Tables

**Table 1:** Physical characteristics of the participants (*n* = 26).

Parameter (Unit)	Mean ± Standard Deviation
Age (years old)	22.3 ± 0.4
Height (cm)	172.8 ± 6.3
Weight (kg)	62.0 ± 2.1
Body mass index (kg/m^2^)	20.7 ± 0.5
Eyesight	Left: 0.9 ± 0.3; right: 0.9 ± 0.3

## Data Availability

The data that support the study findings are available from the corresponding author upon reasonable request.
